# Impact of Efavirenz-, Ritonavir-Boosted Lopinavir-, and Nevirapine-Based Antiretroviral Regimens on the Pharmacokinetics of Lumefantrine and Safety of Artemether-Lumefantrine in Plasmodium falciparum-Negative HIV-Infected Malawian Adults Stabilized on Antiretroviral Therapy

**DOI:** 10.1128/AAC.01162-18

**Published:** 2018-10-24

**Authors:** Clifford G. Banda, Fraction Dzinjalamala, Mavuto Mukaka, Jane Mallewa, Victor Maiden, Dianne J. Terlouw, David G. Lalloo, Saye H. Khoo, Victor Mwapasa

**Affiliations:** a University of Malawi, College of Medicine, Blantyre, Malawi; b Malawi-Liverpool-Wellcome Trust Clinical Research Programme, Blantyre, Malawi; c Oxford Centre for Tropical Medicine and Global Health, Oxford, United Kingdom; d Liverpool School of Tropical Medicine, Liverpool, United Kingdom; e University of Liverpool, Liverpool, United Kingdom; f Mahidol-Oxford Tropical Medicine Research Unit, Bangkok, Thailand

**Keywords:** artemether-lumefantrine, antiretroviral therapy, malaria, antimalarial agents

## Abstract

There is conflicting evidence of the impact of commonly used antiretroviral therapies (ARTs) on the pharmacokinetics of lumefantrine and the safety profile of artemether-lumefantrine. We compared the area under the concentration-time curve from 0 h to 14 days (AUC_0–14 days_) of lumefantrine and the safety profile of artemether-lumefantrine in malaria-negative human immunodeficiency virus (HIV)-infected adults in two steps. In step 1, a half-dose adult course of artemether-lumefantrine was administered as a safety check in four groups (*n* = 6/group): (i) antiretroviral naive, (ii) nevirapine-based ART, (iii) efavirenz-based ART, and (iv) ritonavir-boosted lopinavir-based ART. In step 2, a standard-dose adult course of artemether-lumefantrine was administered to a different cohort in three groups (*n* = 10 to 15/group): (i) antiretroviral naive, (ii) efavirenz-based ART, and (iii) ritonavir-boosted lopinavir-based ART. In step 1, lumefantrine's AUC_0–14 days_ was 53% (95% confidence interval [CI], 0.27 to 0.82) lower in the efavirenz-based ART group than in the ART-naive group and was 2.4 (95% CI, 1.58 to 3.62) and 2.9(95% CI, 1.75 to 4.72) times higher in the nevirapine- and ritonavir-boosted lopinavir groups, respectively. In step 2, lumefantrine's AUC_0–14 days_ was 1.9 (95% CI, 1.26 to 3.00) times higher in the ritonavir-boosted lopinavir group and not significantly different between the efavirenz- and ART-naive groups (0.99 [95% CI, 0.63 to 1.57]). Frequent cases of hematological abnormalities (thrombocytopenia and neutropenia) were observed in the nevirapine group in step 1, leading to a recommendation from the data and safety monitoring board not to include a nevirapine group in step 2. Artemether-lumefantrine was well tolerated in the other groups. The therapeutic implications of these findings need to be evaluated among HIV-malaria-coinfected adults. (This study has been registered at the Pan African Clinical Trials Registry under numbers PACTR2010030001871293 and PACTR2010030001971409.)

## Abstract (precis)

There is conflicting evidence of the impact of commonly used antiretroviral therapies (ARTs) on the pharmacokinetics of lumefantrine and the safety profile of artemether-lumefantrine. We compared the area under the concentration-time curve from 0 h to 14 days (AUC_0–14 days_) of lumefantrine and the safety profile of artemether-lumefantrine in malaria-negative human immunodeficiency virus (HIV)-infected adults in two steps.

## INTRODUCTION

In sub-Saharan Africa (SSA), human immunodeficiency virus (HIV) and Plasmodium falciparum malaria infections are coendemic. HIV infection increases susceptibility to malaria (1–3) and the severity of P. falciparum malaria and reduces the efficacy of some antimalarial drugs (4, 5). To combat these infections, the WHO recommends initiation of antiretroviral therapy (ART) in HIV-positive (HIV^+^) individuals regardless of their CD4 cell counts (6) and prompt use of artemisinin-based combination therapies (ACTs) for malaria-infected individuals (7). The most commonly used ARTs in SSA contain nonnucleoside reverse transcriptase inhibitors (NNRTIs) such as efavirenz (EFV) and nevirapine (NVP) or protease inhibitors (PIs) such as ritonavir-boosted lopinavir (LPV/r). Artemether-lumefantrine (AL) is the most widely implemented first-line ACT in the SSA region (3). HIV-malaria coinfection is common in SSA; hence, a large number of HIV^+^ people on ART require concurrent treatment with AL.

Pharmacokinetic (PK) interactions between NNRTI- or PI-containing ART and ACTs are likely since these classes of drugs affect the activity of cytochrome P450 (CYP450) liver enzymes, including CYP3A4 and CYP2B6 (8–11). The interactions may impact the longer-acting partner drug of an ACT, which is vital in preventing posttreatment malaria recrudescence, after the rapid elimination of the artemisinins (12). Previous PK studies have found lower lumefantrine levels in healthy volunteers cotreated with AL and EFV-based ART (EFV-ART) and higher lumefantrine levels in those cotreated with AL and LPV/r-based ART than in those treated with AL only (13–15). However, PK studies on AL and NVP-based ART have produced conflicting results, with some finding higher, lower, or similar lumefantrine levels in HIV^+^ individuals on NVP-based ART compared to those in ART-naive individuals treated with AL only (16–20). Furthermore, few studies have reported the safety profiles of coadministering AL with commonly used antiretroviral drugs in HIV-infected individuals stabilized on ART.

To further characterize the impact of nevirapine-, efavirenz-, or ritonavir-boosted lopinavir-based ART on the PK of lumefantrine and the safety profile of AL, we conducted an intensive PK study to compare secondary PK parameters of lumefantrine and the incidence of treatment-emergent adverse events (AEs) in malaria-negative HIV-infected adults taking AL plus NVP-, EFV-, or LPV/r-based ART or AL only.

## RESULTS

### Characteristics of participants.

In step 1, 26 participants were enrolled in the study; 24 participants were successfully monitored for 28 days. Two participants taking NVP-based ART were discontinued from the study due to protocol deviations and are not included in the analyses. In step 2, 40 of the 43 enrolled study participants completed 28 days of follow-up. Three participants did not have sufficient data points for PK characterization and are not included in the analyses. No participants were enrolled in the NVP arm for step 2 on the advice of the data and safety monitoring board (DSMB) because of the observed hematological abnormalities in step 1. Table S1 in the supplemental material shows the baseline characteristics of participants who completed follow-up in steps 1 and 2. In step 1, the median duration of ART (in months) was significantly longer in the LPV/r group (63.1 months; range, 33.3 to 85.0 months) than in the EFV group (25.1 months; range, 7.8 to 49.3 months) and the NVP group (58.8 months; range, 24.7 to 80.6 months). There were no major differences between baseline characteristics in step 1 or step 2.

### Pharmacokinetics of lumefantrine and interactions with antiretroviral therapy in step 1.

**FIG 1:**
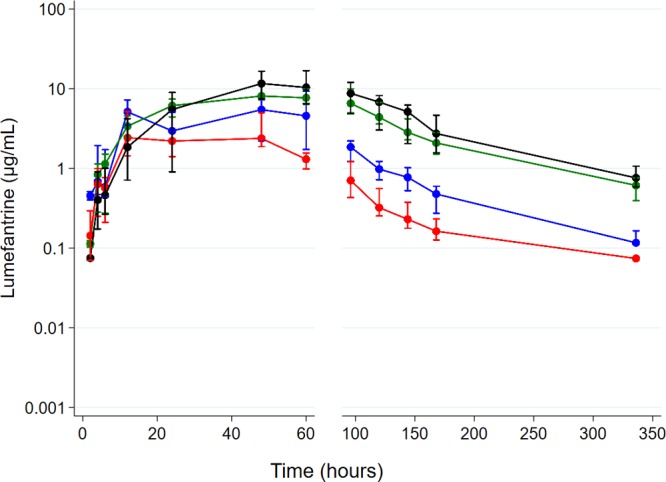
Plasma lumefantrine concentration-time profile in step 1 following administration of half (*n* = 24) the adult treatment course of artemether-lumefantrine among antiretroviral therapy-naive participants (blue) and those on efavirenz (red)-, nevirapine (green)-, and ritonavir-boosted lopinavir (black)-based antiretroviral therapy. Data are presented as medians (interquartile ranges [IQR]).

Table 1 summarizes the PK parameters in the study groups in step 1. Compared with the ART-naive group, the geometric mean area under the concentration-time curve from 0 h to 14 days (AUC_0–14 days_) of lumefantrine was 53% lower in the EFV-ART group, 2.4 times higher in the NVP-ART group, and 2.9 times higher in the LPV/r-based ART group. Similarly, compared with the ART-naive group, lumefantrine's achieved maximum concentration (*C*_max_) was 37% lower in the EFV-ART group, 1.9 times higher in the LPV/r-ART group, and not significantly different in the NVP-based ART arm. Additionally, compared with the ART-naive group, lumefantrine's terminal half-life was 61% shorter in the EFV group but not significantly different in the LPV/r-based and NVP-based ART groups. The median times to reach maximum concentration (*t*_max_) were similar in the NVP-based, EFV-based, and ART-naive groups but slightly longer in the LPV/r-based ART group than in the ART-naive group, with marginal significance. As illustrated in the concentration-time profile in Fig. 1, participants in the LPV/r and NVP-ART groups had higher concentrations of lumefantrine in the terminal elimination phase than those in the ART-naive subgroup, while those in the EFV-based ART group had lower lumefantrine concentrations.

**TABLE 1:** Lumefantrine pharmacokinetic parameters for participants in step 1^*a*^

Parameter	Geometric mean value for study group (95% CI)				Geometric mean ratio for group (95% CI) (*P* value)		
	ART naive (*n* = 6)	NVP (*n* = 6)	LPV/r (*n* = 6)	EFV (*n* = 6)	NVP/ART naive	LPV/r/ART naive	EFV/ART naive
AUC_0–14 days_ (h · μg/ml)	513 (374–703)	1,226 (943–1,594)	1,476 (1,019–2,139)	239 (152–377)	2.39 (1.58–3.62) (0.001)	2.88 (1.75–4.72) (0.001)	0.47 (0.27–0.82) (0.018)
*C*_max_ (μg/ml)	8 (6–10)	12 (8–17)	15 (11–20)	5 (3–7)	1.50 (1.00–2.23) (0.119)	1.88 (1.28–2.68) (0.016)	0.63 (0.36–0.89) (0.054)
*t*_max_ (h)	54 (48–72)	72 (48–72)	72 (72–72)	36 (12–72)	0.295^*b*^	0.060^*b*^	0.365^*b*^
*t*_1/2_ (h)	152 (72–322)	185 (162–212)	223 (171–291)	60 (44–82)	1.22 (0.57–2.62) (0.597)	1.47 (0.66–3.26) (0.341)	0.39 (0.18–0.90) (0.039)

a PK parameters are presented as geometric means (95% confidence intervals), except for *t*_max_ (time to reach maximum concentration) values, which are presented as medians (interquartile ranges). *P* values were calculated using analysis of variance in Stata 15.0 (α = 0.05). NVP, nevirapine-based antiretroviral therapy (ART); EFV, efavirenz-based ART; LPV/r, ritonavir-boosted lopinavir-based ART; AUC_0–14 days_, area under the concentration-time curve from 0 h to 14 days; *C*_max_, achieved maximum concentration; *t*_1/2_, drug elimination half-life.

b *P* value only, calculated using the Wilcoxon rank sum test (α = 0.05).

### Artemether-lumefantrine tolerability and treatment-emergent adverse events in step 1.

AL was well tolerated in all the groups. However, Division of AIDS (DAIDS) grade 3 or 4 treatment-emergent neutropenia was frequently detected across all the study groups: ART naive (3/6 [50.0%]), EFV-based ART (1/6 [16.7%]), LPV/r-based ART (2/6 [33.3%]), and NVP-based ART (3/6 [50.0%]). The intergroup differences were not statistically significant. Additionally, DAIDS grade 3 or 4 treatment-emergent thrombocytopenia was detected in the NVP-based ART group (2/6 [33.3%]) but not in the ART-naive or the LPV/r- and EFV-based ART groups. There was a lack of evidence of a correlation between neutropenia or thrombocytopenia and measured lumefantrine concentrations, and none of these observed adverse events were persistent beyond day 14 of follow-up.

### Pharmacokinetics of lumefantrine and interactions with antiretroviral therapy in step 2.

**FIG 2:**
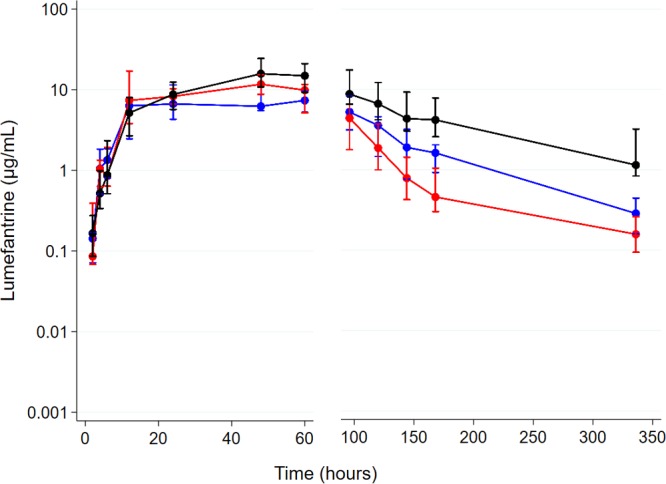
Plasma lumefantrine concentration-time profile in step 2 following administration of the full adult treatment course (*n* = 40) of artemether-lumefantrine among antiretroviral therapy-naive individuals (blue) and those on efavirenz (red)- and ritonavir-boosted lopinavir (black)-based antiretroviral therapy. Data are presented as medians (IQR).

Table 2 summarizes the PK parameters in the study groups in step 2. The geometric mean lumefantrine AUC_0–14 days_ values were similar in the EFV-based ART group and the ART-naive group. Participants in the LPV/r-based ART group had an approximately 1.9-times-higher geometric mean AUC_0–14 days_ than those in the ART-naive group. There were no significant differences in *C*_max_, drug elimination half-life (*t*_1/2_), and median *t*_max_ in the EFV- and LPV/r-based ART groups compared to the ART-naive group. As illustrated in the concentration-time profile in Fig. 2, lumefantrine concentrations were higher in the LPV/r-based ART than in the ART-naive group and were persistently lower in the terminal elimination phase (after 72 h) in the EFV-based ART group than in the ART-naive group.

**TABLE 2:** Lumefantrine pharmacokinetic parameters for participants in step 2^*a*^

Parameter	Geometric mean value for study group (95% CI)			Geometric mean ratio (95% CI) (*P* value)	
	ART naive (*n* = 10)	LPV/r (*n* = 15)	EFV (*n* = 15)	LPV/r/ART naive	EFV/ART naive
AUC_0–14 days_ (h · μg/ml)	1,084 (760–1,547)	2,107 (1,654–2,686)	1,081 (816–1,432)	1.94 (1.26–3.00) (0.004)	0.99 (0.63–1.57) (0.991)
*C*_max_ (μg/ml)	15 (10–23)	19 (16–23)	18 (14–23)	1.27 (0.81–1.93) (0.265)	1.20 (0.75–1.84) (0.456)
*t*_max_ (h)	66 (24–72)	72 (60–72)	48 (12–72)	0.145^*b*^	0.340^*b*^
*t*_1/2_ (h)	160 (103–248)	190 (154–236)	102 (61–170)	1.19 (0.73–1.94) (0.438)	0.64 (0.32–1.26) (0.217)
*C*_d7_ (μg/ml)	1 (0.9–2)	4 (3–6)	0.5 (0.3–0.8)	4.00 (1.72–5.39) (<0.001)	0.50 (0.21–0.74) (0.009)

a PK parameters are presented as geometric means (95% confidence intervals), except for *t*_max_ values, which are presented as medians (interquartile ranges). *P* values were calculated using analysis of variance in Stata 15.0 (α = 0.05). *C*_d7_, day 7 plasma lumefantrine concentration.

b *P* value only, calculated using the Wilcoxon rank sum test (α = 0.05).

### Day 7 lumefantrine concentrations in step 2.

Upon administration of a full standard AL dose, the day 7 mean lumefantrine concentration was 50% lower in the EFV-based ART group than in the ART-naive group. Participants in the LPV/r-based ART group had 4-times-higher day 7 lumefantrine concentrations than those in the ART-naive group, as shown in Table 2. However, the proportions of participants with day 7 lumefantrine concentrations of ≥0.2 μg/ml (200 ng/ml) were not significantly different in the ART-naive group (100% [10/10]), the LPV/r-based ART group (100% [15/15]), and the EFV-based ART group (86.7% [13/15]).

### Artemether-lumefantrine tolerability and treatment-emergent adverse events in step 2.

AL was well tolerated in the three study groups: no DAIDS grade 3 or 4 hematological abnormalities (neutropenia or thrombocytopenia) were reported across the groups. On day 3, corrected QT (QTc) prolongation (>450 ms) was observed in 1 participant in the EFV-based ART group and another in the ART-naive group but in none of the participants in the LPV/r-ART group. All cases resolved by day 7.

## DISCUSSION

This study found that, when treated with a half-dose adult course of AL, individuals on the EFV-based ART regimen had lower lumefantrine exposure (AUC_0–14 days_) than ART-naive individuals, while those in the NVP- or LPV/r-based ART groups had higher AUC_0–14 days_. Similarly, compared to the ART-naive group, *C*_max_ was lower in the EFV-based ART group, higher in the LPV/r-based ART group, and similar in the NVP-based ART group. There were no differences in *t*_max_ across the study groups. The terminal half-life values were significantly lower in the EFV-based ART group but similar in the LPV/r- or NVP-based ART groups compared to those in the ART-naive group. DAIDS grade 3 or 4 treatment-emergent thrombocytopenia and neutropenia were observed upon coadministration of AL and NVP-based ART. When treated with a standard-dose adult course of AL, there was no statistically significant difference in lumefantrine AUC_0–14 days_ between the EFV-based ART group and the ART-naive group, but those on LPV/r-based ART had higher AUC_0–14 days_ than the ART-naive group. There were no significant differences in terminal half-life, *C*_max_, and *t*_max_ between the ART groups and the ART-naive group. Additionally, AL was well tolerated across all study groups.

Our finding, in both steps, of higher lumefantrine exposure (AUC_0–14 days_) and *C*_max_ in the LPV/r-based ART group is consistent with what is known about ritonavir-boosted lopinavir inhibition of CYP450 enzymes (CYP3A4), resulting in higher plasma lumefantrine concentrations since lumefantrine is metabolized by this enzyme entity (13, 14, 21). The therapeutic implications of this observation were previously shown among Ugandan children who had a reduced incidence of malaria when taking lumefantrine and lopinavir-based ART compared to those on NNRTI-based ART (15).

Unlike in step 1, where lumefantrine exposure in the EFV-based ART group was significantly lower than in the ART-naive group, overall lumefantrine exposures (AUC_0–14 days_) in step 2 were surprisingly not significantly different between the two groups. Lumefantrine concentrations in the terminal elimination phase, however, were consistently lower in the EFV-based ART group than in the ART-naive group in both steps (Fig. 1). Since EFV is a known inducer of CYP3A4 enzymes (9), lower lumefantrine concentrations were expected in the terminal elimination phase. The difference in lumefantrine exposures in the EFV-ART/ART-naive comparison could be a result of the use of a parallel-group study design, which is more prone to effects of interindividual anthropometric and genetic variations in CYP450 enzymes than a crossover design. Genetic polymorphisms in CYP450 enzymes are known to impact exposure of drugs metabolized by this enzyme entity (22, 23). Nevertheless, the lower lumefantrine concentrations in the elimination phase among participants on efavirenz-based ART in step 2 are consistent with previous observations (24).

There are conflicting reported results on the PK interactions between AL and NVP-ART, with studies suggesting higher (16, 25), lower (18, 21), or similar (17, 19) lumefantrine exposures in those on AL and NVP-based ART compared to those in individuals on AL alone. This heterogeneity potentially points to genetic variations in CYP activity across settings where HIV-malaria coinfection is endemic. We found higher concentrations of lumefantrine in the NVP-based ART group in step 1 than in the ART-naive group, consistent with findings from a previous study in South Africa (16) and another study conducted in Malawi and Uganda (25). There is evidence that NVP may increase exposure of other drugs metabolized by CYP3A4, as shown by increased *C*_max_ and AUC of darunavir (26) and maraviroc (27), when coadministered with nevirapine, possibly due to reduced metabolism secondary to competitive inhibition of metabolic enzymes (28) or as a result of variations in the availability of proteins to transport drugs (29). Thus, the increased AUC_0–14 days_ and *C*_max_ of lumefantrine in the NVP-based ART group could suggest reduced CYP3A4-mediated metabolism or an unavailability of proteins to transport lumefantrine. Alternatively, the higher exposure of lumefantrine in the NVP-based ART group could be due to potential distinctive inhibition of CYP isoenzymes, such as CYP2C9/19, by NVP, which could be different from that exhibited by other NNRTIs (e.g., EFV). This phenomenon, of drug-specific compared to class-specific inhibition of liver metabolic enzymes by ART, has been previously shown in animal models when ART was coadministered with gliclazide (30).

Neutropenia has been previously documented when ACTs such as artesunate-amodiaquine were administered to HIV-infected children in Uganda (31). In addition, NVP is associated with granulocytopenia as a marker of hypersensitivity (32), but its role in causing thrombocytopenia has not been described. Thus, it is possible that neutropenia could occur following coadministration of NVP and lumefantrine as a result of increased lumefantrine concentrations, increased NVP concentrations, or a synergistic effect of lumefantrine and NVP. In our study population, the occurrence of cases of grade 3 or 4 neutropenia across all study groups in step 1, which were not observed at higher doses in step 2, is likely idiosyncratic since cases of asymptomatic neutropenia have also been previously observed in healthy Malawian adult blood donors (33). Apart from the underlying HIV infection, and with the exception of those on LPV/r-based ART who took it together with zidovudine-ART, none of the participants who experienced thrombocytopenia had other baseline predisposing factors, such as low immunity (CD4 count of <500 cells/mm^3^) or low platelet count. Furthermore, no previous studies have found an association between NVP and thrombocytopenia. The finding of thrombocytopenia in the group receiving AL and NVP is therefore surprising and could be due to chance. Nevertheless, the data and safety monitoring board recommended against administration of a standard-dose adult course of AL with NVP due to the frequent occurrence of thrombocytopenia in addition to neutropenia in the NVP group compared to the ART-naive group in step 1. We therefore were unable to investigate the effect of coadministration of a standard-dose adult course of AL and NVP on the incidence of thrombocytopenia.

Day 7 lumefantrine concentrations are considered to be one of the most important predictors of treatment outcomes following malaria treatment (34, 35). Various investigators have suggested different day 7 lumefantrine cutoffs (36–44), and in a pooled analysis, the Worldwide Antimalarial Resistance Network (WWARN) observed that day 7 lumefantrine concentrations of ≥0.2 μg/ml (200 ng/ml) were associated with a 98% cure rate in uncomplicated malaria patients (parasitemia of <135,000 parasites/μl) (45). In step 2 of this study, although participants on EFV-ART had lower day 7 lumefantrine concentrations than ART-naive participants, and those on LPV/r-based ART had higher concentrations, the proportion achieving lumefantrine concentrations of ≥0.2 μg/ml was only slightly lower in the EFV-ART group but was not significantly different from that in the ART-naive group. This suggests that AL is still likely to be highly efficacious in those on EFV-based ART, despite the PK interaction.

In this study, we did not assess the impact of ART on plasma concentrations of the artemisinin derivatives (artemether and its metabolite dihydroartemisinin), which have a shorter half-life and are crucial in clearing malaria parasites in the early phases of malaria treatment, because we were interested in the longer-acting drug lumefantrine, which confers protection against recrudescence following malaria infection (36, 46). Additionally, we did not quantify NVP plasma concentrations and were not able to assess any potential effect of lumefantrine on the steady-state concentration changes of NVP as well as the subsequent impact on hematological changes. Other limitations include the lack of participant randomization during enrollment and the potential for unmeasured confounders, which may have influenced the observed lumefantrine kinetics. Although the present study had a small sample size, it is unlikely to have missed large (>2-fold) clinically important differences in AUC across the study arms. Furthermore, this study was not designed to elucidate the mechanism of interaction between lumefantrine and ART. Future studies should aim to define these mechanisms, including the role of genetic variations in CYP450 isoenzyme activity, the impact of ART on plasma concentrations of artemisinin derivatives, and the subsequent implication for clearance of malaria parasites among HIV-malaria-coinfected individuals.

In conclusion, we confirmed that coadministration of AL with ritonavir-boosted lopinavir-based antiretroviral therapy resulted in increased lumefantrine exposure, while coadministration of AL with EFV-based ART was associated with lower lumefantrine concentrations, particularly in the terminal elimination phase. Coadministration of AL and NVP-ART was associated with higher lumefantrine exposure and hematological abnormalities (thrombocytopenia and neutropenia) with a half-dose adult course of AL. The therapeutic implications of these findings need to be evaluated in programmatic settings among malaria- and human immunodeficiency virus-coinfected individuals.

## MATERIALS AND METHODS

### Study design.

An open-label, sequential-group PK study was conducted from August 2010 to March 2013 at Queen Elizabeth Central Hospital in Blantyre, Malawi. The study was implemented in the following two steps. In step 1 (Pan African Clinical Trials Registry number PACTR2010030001871293), a half adult dose of AL (2 tablets of AL [Coartem; Novartis], with each tablet containing 20 mg/120 mg of artemether-lumefantrine) was administered at 0, 8, 24, 36, 48, and 60 h to malaria-negative HIV^+^ individuals in the following groups: (i) an antiretroviral-naive (control) group and those receiving (i) NVP-based ART, (iii) EFV-based ART, and (iv) LPV/r-based ART. This step served mainly as a preliminary safety evaluation, checking for unexpected clinical toxicities or interactions.

In step 2 (Pan African Clinical Trials Registry number PACTR2010030001971409), after review of safety data from step 1 by an independent data and safety monitoring board (DSMB), a full standard dose of AL (4 tablets of Coartem, with each tablet containing 20 mg/120 mg AL) was administered at 0, 8, 24, 36, 48, and 60 h to a separate cohort of malaria-negative HIV^+^ individuals in the following groups: (i) an antiretroviral-naive (control) group and those receiving (ii) EFV-based ART and (iii) LPV/r-based ART. The DSMB recommended that step 2 should not proceed with a NVP-based ART group because of safety concerns.

To maximize the absorption of lumefantrine, AL was given with ∼40 ml of soya milk, containing an equivalent of 1.2 g of fat. The first dose of AL in ART participants was timed to coincide with the next scheduled dose of the antiretroviral drugs.

### Study population.

The study population for step 1 and step 2 included HIV-infected male and nonpregnant female adults aged ≥18 years residing in Blantyre, Malawi, or the neighboring districts of Thyolo and Chiradzulu. Individuals on ART were eligible to participate if they had been on NNRTI- or PI-based ART for ≥6 months and had CD4 cell counts of ≥250 cells/mm^3^. At the beginning of the study, HIV-infected antiretroviral-naive individuals were eligible if they had CD4 cell counts of ≥250 cells/mm^3^, but this cutoff point was changed to ≥350 cells/mm^3^ when the WHO criteria for ART initiation changed in July 2011. Other inclusion criteria were body weight of ≥40 kg and willingness to be admitted in the hospital for 3 days, to remain within the study sites, and to be contacted at home or by phone during the course of the study.

We excluded subjects who had a body mass index of <18.5 kg/m^2^; a hemoglobin concentration of <10 g/dl (subsequently changed to <8.5 g/dl based on DSMB recommendation); reported use of any antimalarial drugs within the preceding 4 weeks; reported hypersensitivity to any of the ACTs; receipt of other drugs which are known inhibitors or inducers of P450 enzymes or P-glycoprotein (except co-trimoxazole prophylaxis, which was the standard of care for HIV-infected individuals); a history of regular intake of alcohol (more than twice per week), tobacco (>3 times/week), or any use of illicit drugs; a history or evidence of preexisting liver, kidney, or heart disease, including conductive abnormalities on electrocardiographs (ECGs) (QTc intervals of >450 ms in men and >470 ms in females); clinical and/or laboratory evidence of P. falciparum malaria, hepatitis B, pneumonia, tuberculosis, or bacteremia; laboratory evidence of potentially life-threatening white blood cell disorders, such as an absolute neutrophil count of <0.500 × 10^9^ cells/liter, an absolute lymphocyte count of <0.35 × 10^9^ cells/liter, or an absolute platelet count of <25 × 10^9^ cells/liter; a Karnofsky score of <80%; or concurrent participation in any other clinical trial.

### Sample size.

The sample size in step 1 was 6 in each of the AL/ART and control (ART-naive) groups, and this was based on standard practice in early PK studies of antimalarial drugs, which aimed to safeguard the safety of study subjects and minimize the number of subjects who may be potentially exposed to harmful drug levels. In step 2, the sample size was 15 per group, which had at least 90% power to detect a 2-fold increase in the lumefantrine AUC in any of the AL/ART groups compared with the ART-naive group, assuming a mean (standard deviation) lumefantrine AUC of 0.561 (0.36) μg/ml/h (15) in the ART-naive group, at the level of significance of 5%.

### Ethics and screening procedures.

The design and timing of trial procedures were approved by the College of Medicine Research Ethics Committee (COMREC) in Blantyre, Malawi. The study conformed to the principles of the International Conference on Harmonization on Good Clinical Practice. Research nurses and clinicians sought written informed consent from individuals to perform screening procedures, including physical, medical, and anthropometric assessment; ECGs; and blood tests to detect bloodborne infections and hematological, renal, or hepatic abnormalities. Results from screening procedures were available within 7 days of screening. Based on these results, potential study participants were informed of their eligibility to participate in the study. Thereafter, research nurses or clinicians sought written informed consent from eligible subjects to participate in the study.

### Predosing procedures.

Consenting study participants were reassessed by research nurses or clinicians to determine whether they still met all eligibility criteria, through repeat history taking and physical examination. Eligible participants were admitted in the hospital, and an indwelling cannula was inserted into a vein before their scheduled dose of ART and the first dose of the ACT. Approximately 1 h before the scheduled time of ART and ACT dosing, blood samples were collected for hematological, renal, and liver function tests and a random glucose test.

### Blood sample collection and processing.

During hospitalization, blood samples for PK assays were collected in heparin tubes before treatment and at 1, 2, 4, 6, 12, 24, 36, 48, 60, and 72 h posttreatment. After discharge, blood samples were taken at 4, 5, 6, 7, and 14 days. Immediately after collection, the blood samples were spun in a refrigerated centrifuge, and the separated plasma samples were temporarily frozen in liquid nitrogen before being transferred to a −80°C freezer until PK analyses.

### Safety assessments.

After the first dose of AL, blood samples were collected to detect hematological, renal, and liver function abnormalities at 12, 48, and 72 h and on days 7, 14, 21, and 28. In addition, in step 2, 12-lead electrocardiographs were performed predosing, 5 h after the first dose, and 5 h after the last dose to determine the QTc interval using the Fridericia QT correction formula (47). The study focused on treatment-emergent adverse events (AEs), defined as any clinical or subclinical abnormalities which were absent before dosing with AL but emerged postdosing or those which were present before dosing with AL but worsened postdosing. The severity of AEs was graded using DAIDS criteria (48), while seriousness was defined according to the standard definition.

### Pharmacokinetic assays.

Plasma samples were analyzed for lumefantrine levels at the Malawi-Liverpool-Wellcome Trust Clinical Research Programme in Blantyre, Malawi, using a validated high-performance liquid chromatography (HPLC)-UV assay adopted and transferred to Malawi from the Liverpool School of Tropical Medicine. The PK laboratory in Blantyre participated in WWARN′s external quality assurance program (49). Briefly, lumefantrine and the internal standard (IS) (halofantrine) were recovered from plasma using a single protein precipitation step with acetonitrile and acetic acid (99:1). The supernatant was then evaporated to dryness in a vacuum concentrator at 25°C. The dried extract was redissolved in the reconstitution solvent methanol–0.01 M hydrochloric acid (70:30), and 75 μl was injected into the chromatograph (Agilent 1100). Quantitation of the drugs was achieved by reverse-phase HPLC. The optimum detection wavelength for each drug was 335 nm. The lower limit of quantification (LLQ) of the HPLC-UV assay was 0.05 μg/ml for lumefantrine, with a percent coefficient of variation of <10. Extracted plasma pharmacokinetic samples were run in batches comprising all samples collected from each of any two study participants. Each batch run included a blank plasma extract, two sets of 8-concentration-level calibration standards, and quality controls (QCs) at three concentration levels: low, medium, and high (0.05, 10, and 15 μg/ml). For the batch assay to pass, the measured concentrations of at least 67% of the QC samples had to be within ±20% of their nominal value, and at least one QC had to be acceptable at the LLQ. The mean interassay precision values for low, medium, and high QCs were 6.6%, 8.8%, and 9.2%, respectively. In addition, 75% of each calibration curve's concentrations had to lie within ±20% and ±15% of the nominal concentration at the LLQ or all other concentrations, respectively.

### Data analyses.

Plasma concentrations of lumefantrine were analyzed using noncompartmental pharmacokinetic analysis (NCA), employing the trapezoidal rule with cubic splines. Observed lumefantrine concentrations below the lower limit of quantification (<LLQ) were treated as missing data, except for the predose lumefantrine concentration, which was imputed to zero if below the LLQ. For each study participant, the following PK parameters were computed: AUC_0–14 days_, maximum concentration (*C*_max_), time to maximum concentration (*t*_max_), and terminal elimination half-life (*t*_1/2_). We used Stata 15.0 for the NCA and to compare log-transformed PK parameters. Geometric mean ratios with 95% confidence intervals (CI) are presented. To test for significant differences in PK parameters between each ACT/ART group and the ART-naive group, parametric evaluation of the log-transformed PK parameters was done using analysis of variance (ANOVA) (α = 0.05). Fisher's exact test was used to compare proportions of participants across the study groups with day 7 concentrations that were above a value known to predict treatment response by day 28 and of safety parameters across the different ACT/ART groups in comparison to the ART-naive group. Data summaries and graphics were all performed in Stata 15.0.

## Supplementary Material

Supplemental file 1
