# Supplementary material for: Impact of Efavirenz-, Ritonavir-Boosted Lopinavir-, and Nevirapine-Based Antiretroviral Regimens on the Pharmacokinetics of Lumefantrine and Safety of Artemether-Lumefantrine in Plasmodium falciparum-Negative HIV-Infected Malawian Adults Stabilized on Antiretroviral Therapy
Source: Antimicrob Agents Chemother. 2018 Oct 24;62(11):e01162-18. doi: 10.1128/AAC.01162-18 (PMC6201074; doi:10.1128/AAC.01162-18)
Supplement: Supplemental file 1 [file zac011187584s1.pdf]

Table 1: Baseline Characteristics for study participants in Steps 1 and 2

Characteristic	Step 1					Step 2			
	AL+NVP Containing ART N=6	AL+EFV Containing ART N=6	AL+LPV/r Containing ART N=6	AL without ART N=6	P-value	AL+EFV Containing ART n=15	AL+LPV/r Containing ART n=15	AL without ART n=10	P-value
Gender (n, % female)	4 (67)	3 (50)	4 (67)	4 (67)	1.000	13 (86.7)	10 (66.7)	9 (90.0)	0.402
Median age (range, years)	40 (28-60)	40 (31-48)	43 (34-47)	37 (21-47)	0.851	44 (33-73)	42 (19-70)	32 (24-47)	0.070
Mean haemoglobin (SD, g/dL)	13.3 (2.0)	13.8 (1.7)	13.5 (1.5)	13.0 (1.6)	0.873	12.9 (2.4)	12.4(1.9)	13.1 (1.9)	0.662
Median Body Mass Index (range in kg/m^2)	22.8 (19.3-25.3)	18.9 (17.2-27.5)	23.4 (20.8-37.5)	21.6 (18.8-24.5)	0.102	21.4 (19.2-35.5)	20.5 (17.9-29.4)	21.3 (18.4-27.4)	0.511
Median (range) duration of ART intake at the time of screening (in months)	58.8 (24.7-80.6)	25.1 (7.8-49.3)	63.1 (33.3-85.0)	NA	0.020	77.0 (19.2-103.1)	60.4 (12.5-121.6)	NA	0.854
On Cotrimoxazole prophylaxis, n (%)	6 (100.0)	6 (100.0)	6 (100.0)	6 (100.0)	1.000	10 (66.7)	11 (73.3)	10 (100.0)	0.123
ALT (IU/L)	21.5 (12-44)	23 (12-40)	18 (14-29)	33 (16-55)	0.421	20 (4-31)	19 (12-36)	15.5 (6-44)	0.280
% with AST >ULN n (%)	1 (17)	2 (33)	1 (17)	4 (67)	0.373	4 (26.7)	4 (26.7)	4 (40.0)	0.770
AST (IU/L)	25.5 (13-45)	29.5 (21-48)	28.5 (24-39)	41.5 (25-59)	0.172	26 (20-34)	32 (17-37)	28 (20-63)	0.513

% with ALT >ULN n (%)	2 (33)	1 (17)	0 (0)	3 (50)	0.411	3 (20.0)	3 (20.0)	1 (10.0)	0.770
Alkaline Phosphatase (IU/L)	55 (53-112)	65.5 (44-84)	89.5 (67-165)	54 (41-79)	0.080	74 (28-127)	81 (44-157)	55 (43-86)	0.072
Creatinine (umol/L)	66 (47-87)	50.5 (38-68)	62 (51-89)	65.5 (37-90)	0.421	63 (33-84)	56 (34-91)	62.5 (46-77)	0.962
% with Creatinine >ULN n (%)	0 (0)	0 (0.0)	0 (0)	0 (0)	1.000	0 (0.0)	0 (0.0)	0 (0.0)	1.000
Any anaemia, n (%)	0 (0.0)	0 (0.0)	0 (0.0)	0 (0.0)	1.000	0 (0.0)	4 (26.7)	1 (10.0)	0.100
Any leukopenia, n (%)	0 (0.0)	0 (0.0)	1 (16.7)	0 (0.0)	1.000	3 (20.0)	2 (13.3)	1 (10.0)	0.872
Any neutropenia, n (%)	2 (33.3)	1 (16.7)	2 (33.3)	3 (50.0)	0.932	1 (6.7)	6 (40.0)	4 (40.0)	0.091
Any thrombocytopenia, n (%)	2 (33.3)	0 (0.0)	1 (16.7)	0 (0.0)	0.571	2 (13.3)	6 (40.0)	0 (0.0)	0.064
Median CD4 cell count (range, cells/UL)	526 (317-820)	427 (366-652)	565 (269-670)	356 (254-570)	0.231	458(258-1435)	453(251-1047)	648(354-955)	0.552
